# Reduction of routine radiographs in the follow‐up of distal radius and ankle fractures: Barriers and facilitators perceived by orthopaedic trauma surgeons

**DOI:** 10.1111/jep.13053

**Published:** 2018-11-28

**Authors:** Pieter van Gerven, Leti van Bodegom‐Vos, Nikki L. Weil, Jasper van den Berg, Sidney M. Rubinstein, Marco F. Termaat, Pieta Krijnen, Maurits W. van Tulder, Inger B. Schipper

**Affiliations:** ^1^ Department of Traumasurgery Leiden University Medical Center Leiden The Netherlands; ^2^ Department of Medical Decision Making Leiden University Medical Center Leiden The Netherlands; ^3^ Department of Health Sciences, Faculty of Science, Amsterdam Public Health research institute Vrije Universiteit Amsterdam Amsterdam The Netherlands

**Keywords:** ankle fractures, barriers and facilitators, choosing wisely, de‐implementation, distal radius fractures, radiography

## Abstract

**Rationale, aims, and objectives:**

Studies suggest that routine radiographs during follow‐up of distal radius and ankle fractures result in increased radiation exposure and health care costs, without influencing treatment strategies. Encouraging clinicians to omit these routine radiographs is challenging, and little is known about barriers and facilitators that influence this omission. Therefore, this study aims to identify barriers and facilitators among orthopaedic trauma surgeons that might prove valuable towards the design of a deimplementation strategy.

**Methods:**

A mixed‐method approach was used. First, interviews were conducted with orthopaedic trauma surgeons and patients (n = 16). Subsequently, a questionnaire was developed. This questionnaire was presented to 228 orthopaedic trauma surgeons in the Netherlands. Regression analyses were performed in order to identify which variables were independently associated to the decision to stop performing routine radiographs 6 and 12 weeks after trauma if proven not effective in a large randomized controlled trial.

**Results:**

In total, 130 (57%) respondents completed the questionnaire. Of these, 71% indicated they would stop ordering routine radiographs if they were proven not effective. Three facilitators were independent predictors for the intention to omit routine radiographs: This will “lead to lower health care costs” (Odds Ratio [OR]: 5.38 and 4.38), the need for “incorporation in the regional protocol” (OR: 3.66 and 2.66), and this will “result in time savings for the patient” (OR: 4.84).

**Conclusions:**

We identified three facilitators that could provide backing for a deimplementation strategy aimed at a reduction of routine radiographs for patients with distal radius and ankle fractures.

## 1 INTRODUCTION

Over the past decade, the reduction of low‐value care has become progressively more important to increase the overall quality of health care. One of the driving forces behind this change is the “Choosing Wisely” campaign, which started in 2012 in the United States. Choosing Wisely is committed to reducing the use of diagnostic tests, treatments, and procedures if there is evidence of overuse, potential harm, or significant and unjustifiable costs.^1^ Routine radiography in the postacute follow‐up of distal radius and ankle fractures (ie, after an initial follow‐up period of 4 wk) is an example of diagnostic imaging with questionable value.^2^, ^3^

Distal radius and ankle fractures are common for all ages. The incidence rate is approximately 70 to 160 per 100 000 persons for distal radius fractures and 187 per 100 000 persons for ankle fractures.^4^, ^5^, ^6^, ^7^, ^8^, ^9^ Due to the ageing of the population, incidence rates are expected to increase over the coming decades.^10^ Patients with these fractures present a significant burden to the health care system. In order to allow for optimal functional recovery, conservative and operative fracture treatment options aim to optimize and maintain anatomical reduction until fracture healing occurs.^11^, ^12^, ^13^

Radiographs are used to monitor the position of the fracture fragments or the osteosynthesis material, the alignment of the joint, and the bone‐healing process during the initial phase of follow‐up (ie, the first 3 mo). Additional reasons for the use of radiographs include reassurance of the physician and/or patient and medico‐legal motives.^14^ The frequency and timing of routine radiographs are empirically based. National and international protocols recommend two to four radiographs during the initial phase of follow‐up. Typical moments for radiographs in both ankle and distal radius fracture treatment are 1, 2, 6, and 12 weeks after trauma or operative fixation.^15^, ^16^, ^17^, ^18^

Studies that evaluated the value of postsplinting radiographs and radiographs taken at the first post‐operative outpatient clinic visit after a distal radius fracture suggest that these radiographs do not lead to changes in treatment strategies if they were ordered without a clear clinical indication.^14^, ^19^, ^20^ A prospective randomized controlled trial (RCT)—the WARRIOR‐trial—is currently conducted to confirm the safety and cost‐effectiveness of omitting routine follow‐up radiographs at 6 and 12 weeks among patients with distal radius or ankle fractures.^21^ If the WARRIOR‐trial confirms that omitting (“de‐implementing”) follow‐up radiographs without a clear clinical indication is safe and cost‐effective, this may lay a foundation for a change in the radiographic follow‐up of wrist and ankle fractures. Radiographs taken without a clear clinical indication can then be added to the list of low‐value diagnostic tests that can be consulted at the Choosing Wisely website (www.choosingwisely.org).

When performing a clinical trial, research on how to implement possible findings is an important step. Previous studies have shown that solely publishing trial results that demonstrate the redundancy of a certain treatment or test, or simply publishing Choosing Wisely recommendations, did not typically lead to an abandonment of low‐value care.^22^, ^23^, ^24^ To actually change practice, a strategy is needed to address barriers and facilitators to the change.^25^, ^26^ Currently, detailed insight in barriers and facilitators influencing orthopaedic trauma surgeons to adopt a suggested change in follow‐up protocol of distal radius and ankle fractures is lacking. Therefore, this study aims to identify the specific barriers and facilitators among orthopaedic trauma surgeons for reducing the use of routine radiographs in the follow‐up of distal radius and ankle fractures. We achieved to identify several independently associated facilitators influencing the reduction of routine radiography in the follow‐up of distal radius and ankle fractures.

## 2 METHODS AND MATERIALS

### 2.1 Study design

In this cross‐sectional survey, orthopaedic trauma surgeons in the Netherlands were invited to complete an Internet‐based questionnaire. The Medical Ethics Committee of the Leiden University Medical Center approved the study (protocol number P14.214).

### 2.2 Questionnaire development

To explore potential barriers and facilitators for the de‐implementation of routine radiographs during follow‐up of distal radius and ankle fractures, semistructured interviews were performed with 10 health care professionals (orthopaedic trauma surgeons) and with six patients (three with a distal radius fracture and three with an ankle fracture). Purposive sampling of health care professionals was applied to obtain contrasting views and identify all potentially relevant barriers and facilitators. To increase generalizability orthopaedic trauma surgeons from different regions in the Netherlands, working in university and nonuniversity hospitals was selected. For practical reasons, only patients treated at a single university hospital were asked to participate. They were contacted during their first outpatient clinic visit, or by phone in the week following their first visit.

The frameworks of Grol and Wensing^27^ and Cabana^28^ were used to compose the questions of the semistructured interviews. In both frameworks, barriers and facilitators for behaviour change are grouped in several domains (ie, the innovation itself, the individual professional, the patient, the social context, the organizational context, and the economic and political context). In addition to the barriers and facilitators, the professionals were asked about their current follow‐up protocol for distal radius and ankle fractures. This was done because current usage of radiography might influence the willingness to adopt a protocol using less routine radiography. Both the professionals and the patients were asked for their opinion about a protocol prescribing radiographs at 6 and 12 weeks only on clinical indication.

The interviews were audiotaped, transcribed, and saved anonymously. Subsequently, the transcribed interviews were qualitatively analysed. Two researchers (J.v.d.B. and N.W.) independently marked potential barriers and facilitators. In case of discrepancies, a third researcher (L.v.B.) was consulted. The qualitative analysis was executed using the software package ATLAS.ti (ATLAS.ti Scientific Software Development GmBH, Berlin, Germany). A total of 11 barriers and 15 facilitators were identified during the semistructured interviews. Five items were on the professional level, 10 on the patient level, six on the organizational level, and five on the level of the external environment. No items were identified on the level of innovation or social context. These items were used in the Internet‐based questionnaire for the orthopaedic trauma surgeons.

### 2.3 Survey

#### 2.3.1 Questionnaire

The first part of the questionnaire included questions about demographic characteristics such as age, gender, and years of work experience. In addition, questions were included about the follow‐up protocol for distal radius and ankle fractures currently used by the respondents and the number of patients with a distal radius or ankle fracture they treat annually. The second part of the questionnaire consisted of 26 items covering barriers and facilitators identified from the interviews. The orthopaedic trauma surgeons were asked to what extent they agreed with each barrier or facilitator. Answers could be given on a 4‐point Likert scale with options being: “totally disagree,” “partially disagree,” “partially agree,” and “totally agree.” The third part included questions about the intention to stop performing routine radiographs if these were proven not to be clinically effective in the WARRIOR‐trial. Four response options were given: (a) no; (b) yes, for both distal radius and ankle fractures; (c) yes, but only for distal radius fractures; and (d) yes, but only for ankle fractures.

In a pilot, two local orthopaedic trauma surgeons filled out the questionnaire to test the comprehensibility of the questions and the response categories. No changes to the initial questionnaire were deemed necessary after this assessment.

#### 2.3.2 Population

The developed Internet‐based questionnaire was sent to all surgeons registered with the Dutch Trauma Association (n = 236). Nonresponders received four reminders at 1, 3, 4, and 5 weeks after the first invitation.

### 2.4 Statistical analysis

Data from all respondents who completed the survey were included in the analyses. Descriptive statistics were used to describe baseline characteristics of the respondents and to report the answers to the barrier and facilitator items of the questionnaire.

The data from the questions concerning barriers and facilitators were dichotomized into “disagree” (grouping the answering categories “totally disagree” and “partly disagree”) and “agree” (grouping “totally agree” and “partly agree”) because of little observations in some cells. The values of some baseline characteristics were also dichotomized: The number of years of work experience was dichotomized into “0‐10 years” and “>10 years,” and the annual number of treated patients was dichotomized into “0‐50 patients” and “>50 patients.”

Two groups of respondents were defined: the surgeons who indicated that they intended to stop performing routine radiographic imaging at 6 and 12 weeks after trauma or operative fixation if proven not clinically effective (hereafter referred to as the “intend‐to‐stop” group) and the surgeons who indicated that they did not intended to do so (hereafter referred to as the “intend‐to‐continue” group).

The background characteristics, current usage of radiography, and response to each barrier and facilitator were compared between the intend‐to‐stop and intend to continue groups. Differences between groups were tested with chi‐square. The Fisher exact test was used when the number of observations in a cell was less than 6. These analyses were stratified for distal radius and ankle fractures.

Barriers and facilitators with a statistically significant difference between groups (*P* < 0.05) were considered as potential predictors. Next, as individual barriers and facilitators may be related to others, we included all potential predictors in a multivariate logistic regression model (*P* < 0.05), using a backward stepwise, likelihood ratio method. The intention to stop performing routine radiographs was analysed as the dependent variable, and the barriers and facilitators were analysed as the independent variables. All analyses were performed using IBM SPSS Statistics 23 (IBM Corp, Armonk, NY).

## 3 RESULTS

### 3.1 Respondent characteristics

Of the e‐mail invitations sent to 236 Dutch orthopaedic trauma surgeons, seven failed to be delivered and one surgeon indicated that he or she did not work as an orthopaedic trauma surgeon anymore, resulting in 228 invitations. The questionnaire was completed by 130 orthopaedic trauma surgeons (response rate 57%). The reason for nonresponse was not verified.

Table 1 shows the baseline characteristics of the respondents. The vast majority (95%) were male, and the mean age was 48 years; 55% of the respondents treat over 50 distal radius fractures, and 34% treat over 50 ankle fractures annually. There were no differences in baseline characteristics between the intend‐to‐stop and intend‐to‐continue groups (data not shown).

**Table 1:** Baseline characteristics of respondents

Characteristic	Orthopaedic Trauma Surgeons
	N = 130
Gender	
Male	124 (95%)
Female	6 (5%)
Mean age (SD)	48.3 (8.4)
Work experience	
0‐5 y	22 (17%)
6‐10 y	44 (34%)
11‐15 y	16 (12%)
16‐25 y	32 (25%)
>25 y	16 (12%)
Work environment (multiple options possible)	
University hospital	26 (20%)
Teaching hospital	56 (43%)
General hospital	58 (45%)
Treated patients per year	
Distal radius fractures	
0	1 (1%)
1‐10	1 (1%)
11‐30	25 (19%)
31‐50	31 (24%)
>50	72 (55%)
Ankle fractures	
0	1 (1%)
1‐10	1 (1%)
11‐30	41 (31%)
31‐50	43 (33%)
>50	44 (34%)

In total, 71% of the orthopaedic trauma surgeons had the intention to stop taking radiographs routinely for both distal radius fractures and ankle fractures if these radiographs were proven not to be effective in the Warrior‐trial (Table 2).

**Table 2:** Number of orthopaedic trauma surgeons with the intention to stop taking routine radiographs at weeks 6 and 12 if proven not to be effective in the WARRIOR‐trial

Intention to Stop Taking Routine Radiographs	N = 130
Yes, in distal radius *and* ankle fractures	92 (70.8%)
Yes, in distal radius fractures only	18 (13.8%)
Yes, in ankle fractures only	4 (3.1%)
No	16 (12.3%)

### 3.2 Current radiographic follow‐up

**Figure 1:**
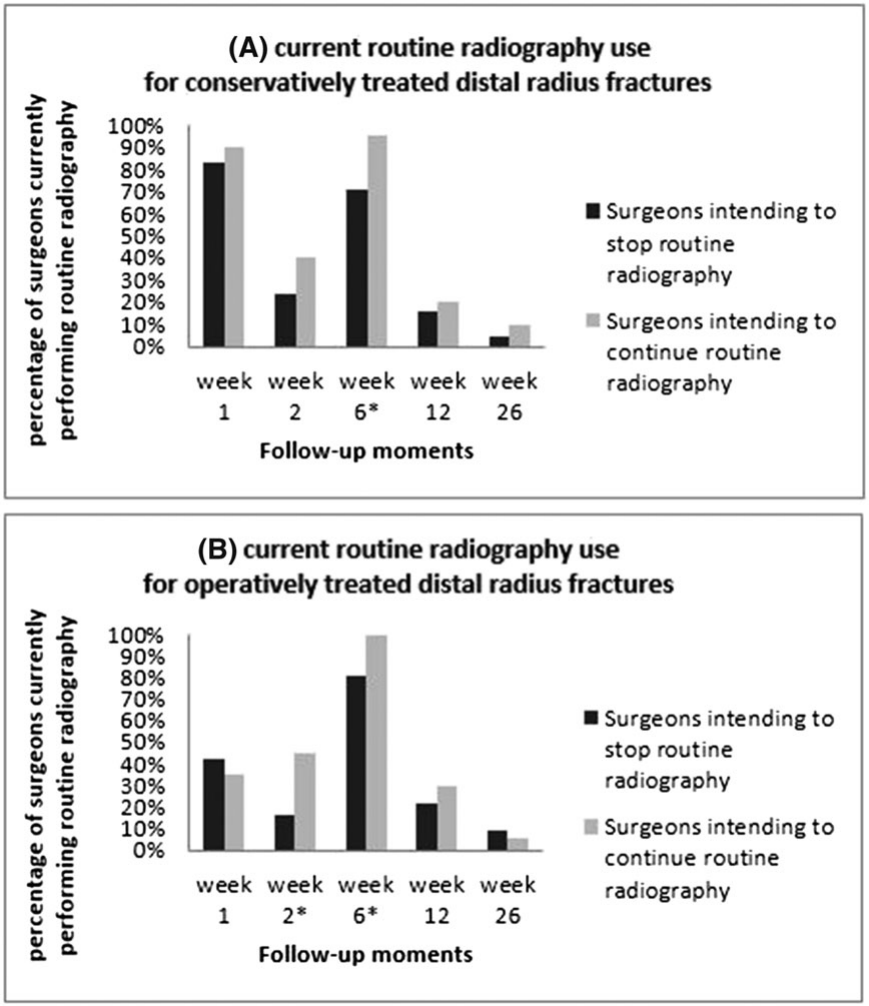
Percentage of surgeons who currently order routine radiographs on specific follow‐up moments for (A) conservatively and (B) operatively treated distal radius fractures, separately for the surgeons who intend to stop or continue ordering routine radiographs if these are proven not to be effective. An asterisk indicates a statistical difference between the surgeon groups for specific follow‐up moments

**Figure 2:**
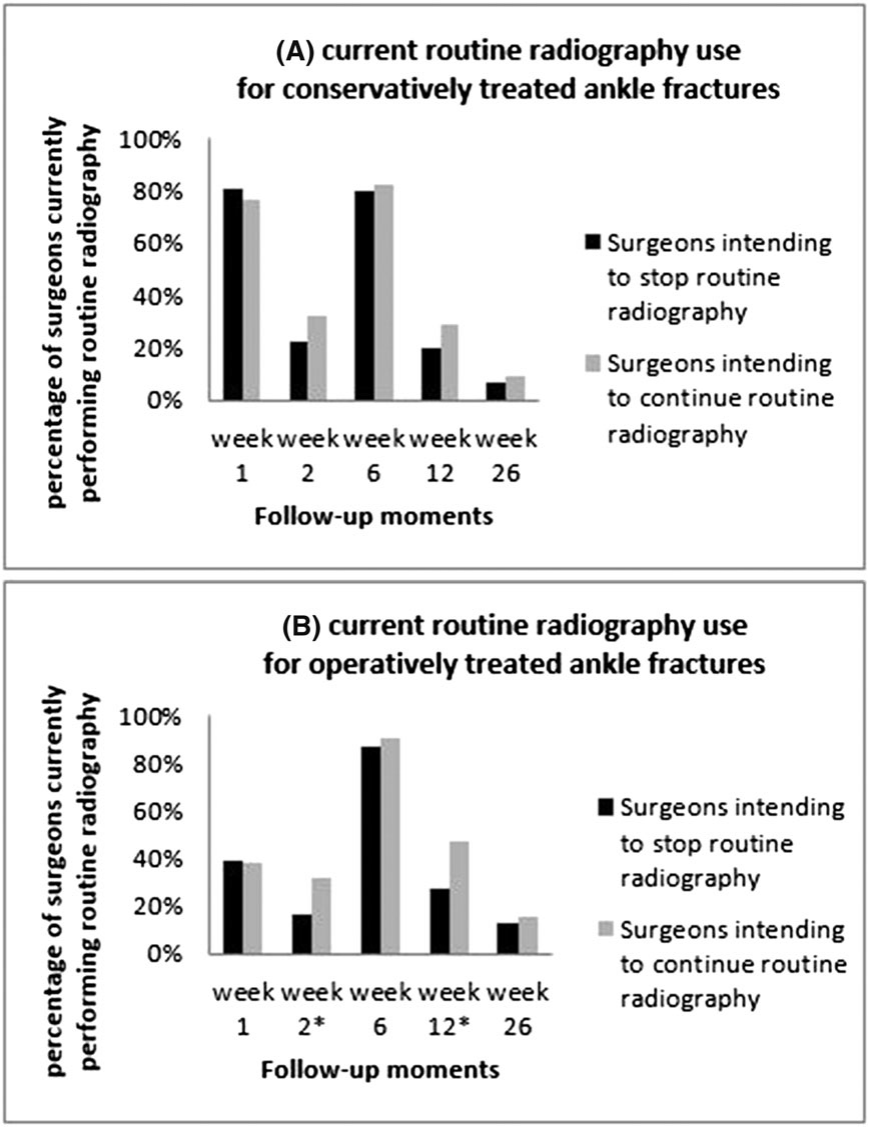
Percentage of surgeons who currently order routine radiographs on specific follow‐up moments for (A) conservatively and (B) operatively treated ankle fractures, separately for the surgeons who intend to stop or continue ordering routine radiographs if these are proven not to be effective. An asterisk indicates a statistical difference between the surgeon groups for specific follow‐up moments

The current radiographic follow‐up strategy used by the responding orthopaedic trauma surgeons for conservatively and operatively treated distal radius fractures is depicted in Figure 1. Results are reported separately for both groups. The current follow‐up strategy for ankle fractures is highlighted in the same manner in Figure 2. Overall, the majority of respondents indicated to order radiographs after approximately 6 weeks for both conservatively (75%) and operatively treated distal radius fractures (84%), as well as for both conservatively (81%) and operatively treated (88%) treated ankle fractures. Respondents from the intend‐to‐stop group were significantly less likely to obtain radiographs as a routine part of their current practice. For distal radius fractures, less radiographs were obtained at 6 weeks when treated conservatively (71% vs 95%, *P* < 0.05). For operatively treated distal radius fractures, less radiographs were obtained at week 2 (16% vs 45%, *P* < 0.05) and week 6 (81% vs 100%, *P* < 0.05). For operatively treated ankle fractures, less radiographs were ordered at week 2 (16% vs 32%, *P* < 0.05) and week 12 (27% vs 47%, *P* < 0.05). At other time points, there were no differences between groups.

### 3.3 The influence of barriers and facilitators

Table 3 shows the barriers and facilitators in the questionnaire for each domain of the framework according to Grol and Wensing and the overall percentages of orthopaedic trauma surgeons who did or did not agree with these barriers and facilitators.

**Table 3:** Agreement with barriers and facilitators among respondents

	Statement, %
The professional	
Follow‐up radiographs of distal radius and ankle fractures around 6 and 12 wk after trauma …	
… are necessary to evaluate the treatment outcome, because I often change my policy based on the radiographs taken at 6 and 12 wk (B)	20.0
... are essential for the surgeon to learn how to interpret radiographs (B)	21.5
… provide me with essential feedback about the treatment outcome (B)	50.0
… provide me with certainty about the treatment outcome (B)	21.5
Not standardly taking radiographs of wrist and ankle fractures at weeks 6 and 12 …	
… leads to a lower workload for the surgeon (F)	32.3
The patient	
Follow‐up radiographs of distal radius and ankle fractures around 6 and 12 wk after trauma …	
… are necessary to evaluate the treatment outcome, because patients do not adequately report their complaints beyond the initial 2‐wk follow‐up (B)	16.2
… are necessary to provide custom care (B)	37.7
… are necessary to make a prognosis (B)	44.6
… are necessary to correctly evaluate the final outcome of the treatment (B)	51.5
… are necessary to evaluate the interim outcome of the treatment, besides other parameters such as function or pain (B)	58.5
… give the patient certainty about the healing process (B)	65.4
Not standardly taking radiographs of distal radius and ankle fractures around weeks 6 and 12 after trauma …	
… leads to significantly less radiation exposure for the patient (F)	42.3
… leads to a cost reduction for the patient (F)	46.9
… results in more patient‐friendly care (F)	57.7
… results in time saving for the patient (F)	79.2
The organizational context	
Not standardly taking radiographs of distal radius and ankle fractures around weeks 6 and 12 after trauma … …	
… is only possible with the support of the plastic surgery department (F)	19.2
… is only possible with the support of the radiology department (F)	21.5
… is only possible with the support of the orthopaedic department (F)	41.5
… leads to less workload in the surgical department (F)	46.9
… leads to less workload in the radiology department (F)	85.4
… results in lower health care costs for the Netherlands (F)	82.3
External environment	
Follow‐up radiographs of distal radius and ankle fractures around 6 and 12 wk after trauma …	
… are necessary for medico‐legal protection (B)	56.2
Not standardly taking radiographs of distal radius and ankle fractures around weeks 6 and 12 after trauma … …	
… is only possible if it is incorporated in the national protocol (F)	43.8
… is only possible if it is incorporated in the regional protocol (F)	46.9
… is only possible if it is incorporated in the local protocol (F)	72.3
No items were on the level of innovation, social context	

Abbreviations: B, barrier; F, facilitator.

The three most frequently perceived barriers for omitting routine radiographs were in the domain of the patient and the domain of the external environment. The statements involved were as follows: follow‐up radiographs of distal radius and ankle fractures at 6 and 12 weeks after trauma “give the patient certainty about the healing process” (65.4% agreement), “are necessary to evaluate the interim outcome of the treatment, besides other parameters such as function or pain” (58.5% agreement), and “are necessary for medico‐legal protection” (56.2% agreement).

The three facilitators that the respondents most frequently agreed with were on the domain of the organizational context and the domain of the patient. They included the statements that not standardly taking radiographs of distal radius and ankle fractures around 6 and 12 weeks after trauma “leads to less pressure on the radiology department” (85.4% agreement), “results in lower health care costs for the Netherlands” (82.3% agreement), and “results in time saving for the patient” (79.2% agreement).

Table 4 shows the percentage of surgeons who agreed with the barriers and facilitators in the questionnaire when grouped to the intention to omit radiographs or not. For distal radius fractures, responses concerning one of the barriers and seven of the facilitators showed a difference between the intend‐to‐stop group and the intend‐to‐continue group. For ankle fractures, responses concerning eight facilitators showed a difference as well. A large degree of overlap existed between the found facilitators in distal radius fractures and ankle fractures.

**Table 4:** Agreement with barriers and facilitators separately for surgeons who intend to stop or continue with ordering routine radiographs, if these are proven not to be effective for distal radius fractures or ankle fractures^a^

	Distal Radius Fractures		Ankle Fractures	
	Stop (n = 110)	Continue (n = 20)	Stop (n = 96)	Continue (n = 34)
The professional				
Follow‐up radiographs of distal radius and ankle fractures around 6 and 12 weeks after trauma …				
… are necessary to evaluate the treatment outcome, because I often change my policy based on the radiographs taken at 6 and 12 weeks (B)	21 (19.1%)	5 (25%)	16 (16.7%)	10 (29.4%)
... are essential for the surgeon to learn how to interpret radiographs (B)	23 (20.9%)	5 (25%)	22 (22.9%)	6 (17.6%)
… provide me with essential feedback about the treatment outcome (B)	**50 (45.5%)**	**15 (75%)**	45 (46.9%)	20 (28.8%)
… provide me with certainty about the treatment outcome(B)	62 (56.4%)	13 (65%)	56 (58.3%)	19 (55.9%)
Not standardly taking radiographs of wrist and ankle fractures at week 6 and 12 weeks …				
… leads to a lower workload for the surgeon (F)	39 (35.5%)	3 (15%)	**37 (38.5%)**	**5 (14.7%)**
The patient				
Follow‐up radiographs of distal radius and ankle fractures around 6 and 12 weeks after trauma …				
… are necessary to evaluate the treatment outcome, because patients do not adequately report their complaints beyond the initial 2‐week follow‐up (B)	17 (15.5%)	4 (20%)	16 (16.7%)	5 (14.7%)
… are necessary to provide custom care (B)	40 (36.4%)	9 (45%)	33 (34.4%)	16 (47.1%)
… are necessary to make a prognosis (B)	47 (42.7%)	11 (55%)	39 (40.6%)	19 (55.9%)
… are necessary to correctly evaluate the final outcome of the treatment (B)	54 (49.1%)	13 (65%)	46 (47.9%)	21 (61.8%)
… are necessary to evaluate the interim outcome of the treatment, besides other parameters such as function or pain (B)	61 (55.5%)	15 (75%)	52 (54.2%)	24 (70.6%)
… give the patient certainty about the healing process (B)	71 (64.5%)	14 (70%)	65 (67.7%)	20 (58.8%)
Not standardly taking radiographs of distal radius and ankle fractures around week 6 and 12 weeks after trauma …				
… leads to significantly less radiation exposure for the patient (F)	**52 (47.3%)**	**3 (15%)**	45 (46.9)	10 (29.4%)
… leads to a cost reduction for the patient (F)	**57 (51.8%)**	**4 (20%)**	**52 (54.2%)**	**9 (26.5%)**
… results in more patient‐friendly care (F)	**70 (63.6%)**	**5 (25%)**	**64 (66.7%)**	**11 (32.4%)**
… results in time saving for the patient (F)	**92 (83.6%)**	**11 (55%)**	**85 (88.5%)**	**18 (52.9%)**
The organizational context				
Not standardly taking radiographs of distal radius and ankle fractures around week 6 and 12 weeks after trauma …				
… is only possible with the support of the plastic surgery department (F)	22 (20.0%)	3 (15%)	20 (20.8%)	5 (14.7%)
… is only possible with the support of the radiology department (F)	23 (20.9%)	5 (25%)	22 (22.9%)	6 (17.6%)
… is only possible with the support of the orthopaedic department (F)	48 (42.6%)	6 (30%)	41 (42.7%)	13 (38.2%)
… leads to less workload in the surgical department (F)	**57 (51.8%)**	**4 (20%)**	**53 (55.2%)**	**8 (23.5%)**
… leads to less workload in the radiology department (F)	97 (88.2%)	14 (70%)	**88 (91.7%)**	**23 (67.6%)**
… results in lower health care costs for the Netherlands (F)	**98 (89.1%)**	**9 (45%)**	**88 (91.7%)**	**19 (55.9%)**
External environment				
Follow‐up radiographs of distal radius and ankle fractures around 6 and 12 weeks after trauma …				
… are necessary for medico‐legal protection (B)	60 (54.5%)	13 (65%)	52 (54.2%)	21 (61.8%)
Not standardly taking radiographs of distal radius and ankle fractures around week 6 and 12 weeks after trauma …				
… is only possible if it is incorporated in the national protocol (F)	49 (44.5%)	8 (40%)	45 (46.9%)	12 (35.3%)
… is only possible if it is incorporated in the regional protocol (F)	**56 (50.9%)**	**5 (25%)**	**50 (52.1%)**	**11 (32.4%)**
… is only possible if it is incorporated in the local protocol (F)	81 (73.6%)	13 (65%)	71 (74.0%)	23 (67.6%)

Abbreviations: B, barrier; F, facilitator.

a Bold numbers indicate a statistical difference between groups (*P* < 0.05).

Based on the univariate analyses (Table 4), one of the barriers and a total of nine facilitators for omitting routine radiography were included in the multivariate logistic regression analyses, predicting the intention to stop performing radiographs at 6 and 12 weeks if proven not to be effective. Table 5 shows that for distal radius fractures, two facilitators remained in the final model and were found to be independently associated with the intention to stop ordering routine radiographs. Respondents from the intend‐to‐stop group were more convinced that not taking routine radiographs will result in lower health care costs for the Netherlands (odds ratio [OR] 5.38, 95% CI, 1.61‐17.99). These respondents were also more likely to value the regional protocols (OR 3.66, 95% CI, 1.08‐12.4). For ankle fractures, three facilitators were found to be independently associated with the intention to omit routine radiography if proved to be not clinically effective. Respondents from the intend‐to‐stop group were more convinced that omitting routine radiography for ankle fractures would lead to lower health care costs as well (OR 4.38, 95% CI, 1.45‐13.28). Moreover, for ankle fractures, these respondents also value the regional protocol more (OR 2.66, 95% CI, 1.01‐6.99). Furthermore, for ankle fracture patients, the facilitator “not standard taking radiographs result in time saving for the patient” was another independent predictor for the intention of omitting routine radiography (OR 4.84, 95% CI, 1.63‐14.37).

**Table 5:** Multivariate logistic regression analysis predicting the intention to stop ordering routine radiographs at 6 and 12 weeks after trauma if proven not effective for distal radius fractures and ankle fractures^a^

	Distal Radius Fractures	Ankle Fractures
The professional		
Follow‐up radiographs of distal radius and ankle fractures around 6 and 12 weeks after trauma …		
… provide me with essential feedback about the treatment outcome (B)	OR 0.38 (95% CI, 0.11‐1.29)	…
Not standardly taking radiographs of wrist and ankle fractures at week 6 and 12 weeks …		
… leads to a lower workload for the surgeon (F)	…	OR 1.09 (95% CI, 0.23‐5.14)
The patient		
Not standardly taking radiographs of distal radius and ankle fractures around week 6 and 12 weeks after trauma …		
… leads to significantly less radiation exposure for the patient (F)	OR 2.20 (95% CI, 0.51‐9.11)	…
… leads to a cost reduction for the patient (F)	OR 1.81 (95% CI, 0.47‐6.95)	OR 1.66 (95% CI, 0.62‐4.50)
… results in more patient‐friendly care (F)	OR 3.33 (95% CI, 0.99‐11.20)	OR 2.25 (95% CI, 0.83‐6.11)
… results in time saving for the patient (F)	OR 1.01 (95% CI, 0.21‐4.76)	**OR 4.84 (95% CI**, **1.63‐14.37)**
The organizational context		
Not standardly taking radiographs of distal radius and ankle fractures around week 6 and 12 weeks after trauma … …		
… leads to less workload in the surgical department (F)	OR 0.679 (95% CI, 0.11‐3.42)	OR 0.96 (95% CI, 0.28‐3.23)
… leads to less workload in the radiology department (F)	…	OR 1.81 (95% CI, 0.49‐6.65)
… results in lower health care costs for the Netherlands (F)	**OR 5.38 (95% CI**, **1.61‐17.99)**	**OR 4.38 (95% CI**, **1.45‐13.28)**
External environment		
Not standardly taking radiographs of distal radius and ankle fractures around week 6 and 12 weeks after trauma …		
… is only possible if it is incorporated in the regional protocol (F)	**OR 3.66 (95% CI**, **1.08‐12.40)**	**OR 2.66 (95% CI**, **1.01‐6.99)**

Abbreviations: B, barrier; CI, confidence interval; F, facilitator; OR, odds ratio.

a Bold numbers indicate a statistical difference between groups (*P* < 0.05).

## 4 DISCUSSION

This study was conducted in order to identify which barriers and facilitators among orthopaedic trauma surgeons influence the abandonment of potential low‐value diagnostic imaging for patients with distal radius and ankle fractures. In this study, multiple barriers and facilitators for reducing low‐value diagnostic imaging were acknowledged by the consulted orthopaedic trauma surgeons. We identified two facilitators that were independently associated with the intention to omit routine radiography in distal radius fracture patients. Three facilitators showed to be of influence on the intention to stop ordering routine radiographs in ankle fracture patients, if the WARRIOR‐trial would prove these routine radiographs to be ineffective. The other reported barriers and facilitators could not be identified to be independently associated with the intended behaviour of the respondents.Two of the aforementioned facilitators showed to be of influence on both distal radius and ankle fracture patients: The notion that reducing the number of radiographs in the follow‐up of distal radius and ankle fracture leads to cost savings for the health care system, and the need of incorporation of the trial's findings in the regional protocol. A future de‐implementation strategy, assuming that the WARRIOR‐trial will provide evidence for the reduction of the number of routine radiographs without compromising the quality of care, should focus on changing the current protocols into protocols with fewer radiographs on a regional level. Besides that, a thorough cost‐effectiveness analysis needs to be performed, in order to confirm the assumption that implementation of such a protocol will lead to a reduction in cost.

To our knowledge, no previous studies on barriers and facilitators for de‐implementation of routine radiographs have been conducted. Voorn et al assessed barriers among orthopaedic surgeons and anaesthesiologists for the intention to stop the use of erythropoietin (EPO) and blood salvage in total hip and total knee arthroplasty.^24^ They found that the intention to stop EPO and blood salvage was related to current blood management protocols, as well as to their own technical skills, patient safety, and a lack of interest to save money. The availability of up‐to‐date protocols and clinical guidelines also plays an important role in implementation. For instance, the framework of Cabana et al^28^ shows that awareness of and familiarity with a protocol or guideline influences the knowledge of the physicians. This is the first requirement for behaviour change. De‐implementation of the routine radiographs in the follow‐up of distal radius and ankle fractures by revising the current protocol could be a first step towards the change in behaviour of surgeons. At the domain of the patient, saving time when no radiograph of the ankle is needed is a facilitator more frequently acknowledged by respondents from the intend‐to‐stop group. In the organizational context, the potential decrease in cost when reducing the number of radiographs might also prove to be a good starting point for omitting this type of low‐value care. From literature, it is known that dissemination of protocols alone is not enough to change behaviour of surgeons. As shown by Prior et al, more educational outreach, such as oral presentation on local, regional, and national levels, is needed to inform the surgeons about the newly incorporated protocol. This kind of outreach is needed to effectively lead to the abandonment of routine radiography at 6 and 12 weeks for distal radius and ankle fractures.^29^ Although four out of six barriers and facilitators perceived most frequently by the orthopaedic trauma surgeons in this study were not independently associated with the intention to stop performing routine radiographs, these barriers and facilitators can still be useful in the educational outreach to inform the surgeons about the revised protocol.

### 4.1 Strengths and limitations

By conducting semistructured interviews, a complete set of barriers and facilitators based on an established framework was provided for the survey, which can be seen as one of the strengths of this study. While orthopaedic trauma surgeons with an interest in development or revision of protocols would have been more likely to participate, it is questionable whether their responses would be any different than those of surgeons who do not have an interest in this area. With a response rate of 57%, which is much higher than the responses found in other surveys among surgeons,^24^, ^30^, ^31^ the chance of response bias is moderate. Additionally, the number of respondents was large (n = 130), further reducing the risk of response bias. The facilitators that were independently associated with the intention to stop performing the routine radiographic imaging are likely to be relevant to convince surgeons to stop performing routine radiographs.

## 5 CONCLUSIONS

Identifying barriers and facilitators among orthopaedic trauma surgeons regarding the use of a protocol with fewer radiographs is crucial for successful de‐implementation of routine radiography for distal radius and ankle fractures. The majority of orthopaedic trauma surgeons intend to follow newly published evidence on the reduced use of routine radiographs. When comparing the intend‐to‐stop and intend‐to‐continue groups, several independently associated facilitators can be identified. The identified facilitators can be of value for the development of a tailored de‐implementation strategy. In this particular case, the strategy should focus on adjusting the current regional protocols into protocols with less routine radiographs and local, regional, and national education. This education should target the potential benefits of the implementation of these protocols in the terms of cost savings and time efficiency. The education on these protocols will also create familiarity with the study outcomes, and a higher awareness among orthopaedic trauma surgeons.
